# Crosstalk between the EGFR and other signalling pathways at the level of the global transcriptional corepressor Groucho/TLE

**DOI:** 10.1038/sj.bjc.6603019

**Published:** 2006-02-28

**Authors:** P Hasson, Z Paroush

**Affiliations:** 1 Division of Developmental Biology, National Institute for Medical Research, Mill Hill, London NW7 1AA, UK; 2 Department of Biochemistry, Faculty of Medicine, The Hebrew University, PO Box 12272, Jerusalem 91120, Israel

**Keywords:** EGFR, Groucho/TLE, Notch, signal transduction, transcriptional regulation

## Abstract

In this minireview, we briefly revisit the *Drosophila* Notch and epidermal growth factor receptor pathways, and relate to the relationship between them. We then mainly focus on the involvement of Groucho (Gro)/TLE, a global developmental corepressor, in these pathways. In particular, we discuss Gro/TLE's role at the junction between these two signal transduction cascades.

A handful of signalling pathways are repeatedly used to generate the innumerable varied cell types that comprise the bodies of all multicellular organisms. Mounting evidence suggests that the genesis of cellular complexity by so few pathways relies to a large extent on the crosstalk between them, that is, on the integration of signals transduced by these cascades, and on their combinatorial activities. For crosstalk to occur, whether with synergistic or antagonistic outcomes, signalling pathways must act on common cellular targets. In the nucleus, for example, diverse signalling pathways often impinge on a given promoter, which thus integrates the multiple cues required for stringent control of that specific gene. Recent findings from *Drosophila* now suggest that inputs from signalling pathways also converge on general transcriptional coregulators, in a way that can alter gene expression programmes involving large arrays of genes. Below, we focus on the antagonism between the Notch (N) and the epidermal growth factor receptor (EGFR) pathways, and on the role played by the universal transcriptional corepressor Groucho (Gro) as a focal point for cross-pathway regulation.

## THE NOTCH PATHWAY

**Figure 1:**
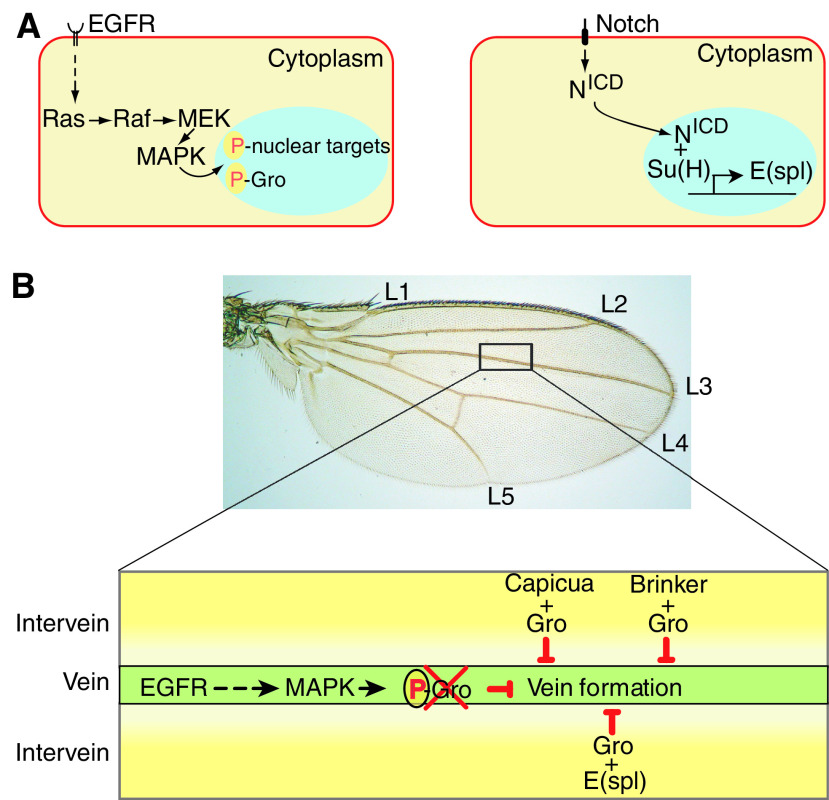
EGFR signalling leads to the formation of wing veins by overriding Groucho-mediated repression. (**A**) Shown are abbreviated presentations of the EGFR and Notch signalling pathways. (**B**) A photograph of a *Drosophila* wing, with the veins numbered (L1–L5). Below, a schematic illustration of adjacent vein and intervein regions, depicting how MAPK phosphorylation of Gro may relieve transcriptional silencing by multiple antivein Gro-dependent repressors, promoting the formation of wing veins.

The highly conserved N signalling pathway participates in multiple developmental processes that entail cell fate determination in both invertebrates and higher organisms. This pathway is perhaps best known for its role in ‘lateral inhibition’, a biological setting in which the N cascade allows a signal emanating from one cell to alter the differentiation state of its initially equivalent neighbours. It thus acts to prevent groups of equipotent cells from all acquiring identical fates, thereby ensuring cellular diversity. In brief, the binding of one of two ligands, Serrate or Delta (Jagged1/2 and Delta1/3/4 in vertebrates, respectively), to the N receptor triggers a proteolytic cleavage of N, releasing its intracellular domain (N^ICD^) and allowing it to shuttle into the nucleus. In the nuclear compartment, N^ICD^ associates with the DNA-binding transcription factor Suppressor of Hairless (Su(H); CBF1/RBP-Jk in vertebrates), and jointly they transduce the N signal by directly activating target gene expression (Figure 1A; Artavanis-Tsakonas *et al*, 1999; Lai, 2004). Prominent among the targets of the Su(H):N^ICD^ complex are the *Enhancer of split* (*E(spl)*) gene products, which are the ultimate executioners of the N signalling cascade and are therefore considered to be its major effectors. The E(spl) proteins (HES in vertebrates) are transcriptional repressors that have been best characterised in the context of neurogenesis as suppressors of proneural gene expression.

## GROUCHO: A NUCLEAR EFFECTOR OF NOTCH AND OTHER SIGNALLING PATHWAYS

Importantly, the E(spl) proteins rely on the general corepressor Gro for silencing transcription of their targets, implicating Gro as a key effector of N signalling (e.g. Paroush *et al*, 1994; Giagtzoglou *et al*, 2003). *Drosophila* Gro is the founding member of a family of highly conserved metazoan corepressors that associate with a myriad of transcription factors, rendering them transcriptional repressors (Chen and Courey, 2000). Notably, the vertebrate homologues of Gro – the *T*ransducin-*L*ike *E*nhancer of split (TLE) and the Gro-*r*elated *g*ene (Grg) proteins – similarly play transcriptional regulatory roles in diverse developmental stages of higher organisms, including in neurogenesis (Fisher and Caudy, 1998; Chen and Courey, 2000).

Interestingly, in the context of the N pathway Gro associates not only with the E(spl) repressors but also with Su(H) itself, allowing the latter to maintain N target genes in a silenced state in the absence of N signalling (Lai, 2004). For lack of space, in this minireview we only focus on Gro's role as a corepressor in conjunction with the E(spl) proteins, and on the developmental settings in which these transcriptional regulators partake.

Significantly, besides being components of the N pathway, the universal Gro/TLE corepressors have also been implicated as effectors of various other signalling cascades. For example, Gro mediates the repressor function of Brinker (Brk) and Tcf/Lef, the nuclear transducers of the Dpp/TGF-*β* and Wingless (Wg)/Wnt signalling pathways, respectively (Brantjes *et al*, 2001; Hasson *et al*, 2001; Zhang *et al*, 2001). Gro-dependent repressors also act downstream of receptor tyrosine kinase (RTK) pathways, for instance those mediated by the Torso receptor and by EGFR (Paroush *et al*, 1997; Price *et al*, 1997). Intriguingly, however, whereas Gro acts as a bona-fide component of the N, Wg/Wnt and Dpp/TGF-*β* pathways, as a coregulator that endows repressor potential on resident transcription factors affiliated to these cascades, it appears to function differently in the context of the EGFR and other RTK signalling pathways, at a novel level of regulation. Below, we assess evidence suggesting that, in effect, Gro-dependent repression is negated by EGFR signalling.

### EGFR SIGNALLING DOWNREGULATES GROUCHO'S REPRESSOR ACTIVITY

The developmental roles played by the EGFR pathway and its involvement in intercellular communication and cell fate specification have been extensively reviewed elsewhere (Shilo, 2005). Briefly, activation of EGFR by its ligands leads to the relay of signals via the generic Ras/Raf/MEK/MAPK cascade, culminating in MAPK phosphorylation. Modified MAPK enters the nucleus and phosphorylates specific target transcription factors, thus linking signalling with gene expression regulation (Figure 1A).

It has lately emerged that Gro is a novel nuclear target for MAPK phosphorylation. Groucho contains two putative, evolutionarily conserved MAPK consensus sites, and is phosphorylated in cell culture and *in vivo* in response to EGFR signalling (Hasson *et al*, 2005). Importantly, the activation of EGFR, or of downstream components of the pathway such as Ras or MAPK, attenuates Gro-mediated repression *in vivo*. Conversely, when either *EGFR* or *Ras* are mutated, an opposite effect is observed, that is, Gro-mediated repression is strengthened (Hasson *et al*, 2005). Consistent with these results, repression is less potent by a derivative of Gro that mimics a constitutively pseudo-phosphorylated form (Gro^DD^), in which the phospho-acceptor residues found in the two MAPK sites have been substituted for by negatively charged Aspartate (D) amino acids. Reciprocally, blocking phosphorylation of the MAPK sites by changing these residues to Alanine (A) (Gro^AA^) leads to the overpotentiation of Gro's corepressor capacity (Hasson *et al*, 2005). Significantly, phosphorylation of Gro in response to MAPK activation hinders the E(spl) proteins – the nuclear effectors of the Notch pathway – from effectively silencing their target genes, as will be described below.

## REGULATION OF GROUCHO-MEDIATED REPRESSION BY PHOSPHORYLATION

Phosphorylation of transcription factors stands out as an effective means by which developmental, mitogenic and metabolic cues alter gene expression in a rapid and reversible manner. Gro/TLE proteins contain putative phosphorylation sites for a number of kinases, and several studies have shown that Gro/TLE-mediated repression is affected by such modifications. Accordingly, Choi *et al* (2005) have recently reported that phosphorylation of Gro by DHIPK2 causes it to dissociate from one of its DNA-binding partners, bringing about derepression of target gene expression. In contrast, Stifani and co-workers have shown that casein kinase 2 phosphorylates Gro/TLE and enhances its repressor capacity, by augmenting its affinity to E(spl) proteins and to chromatin (Nuthall *et al*, 2002). Thus, differential and even opposing effects are brought about as a consequence of Gro/TLE phosphorylation by various kinases, presumably in response to different signals.

The findings assessed here suggest that Gro is also phosphorylated by MAPK, which attenuates its repressor capacity. How does this specific modification affect Gro activity? Theoretically, MAPK phosphorylation could have an impact on one, or even on several, of the many steps leading from the initial recruitment of Gro by DNA-binding partner proteins to, ultimately, transcriptional silencing. To date, little is known about the mechanism by which Gro/TLE proteins, once tethered to target promoters, block transcription of their targets. Clearly, homo-oligomerisation is a prerequisite for Gro/TLE repressor activity and, further, Gro/TLEs physically bind both histones and histone deacetylases (specifically, Rpd3/HDAC1) (e.g. Chen *et al*, 1999; Song *et al*, 2004). Collectively, these observations have led to the following model: Gro is initially tethered to specific gene regulatory regions via its associations with distinct DNA-bound repressors. Subsequently, additional Gro molecules are recruited by means of Gro:Gro and Gro:chromatin interactions. In this way, a complex formed by protein associations between a repressor and Gro may serve as a nucleation centre for regional repressive chromatin, that spreads some distance away from the initial repressor DNA-binding site, and likely accounts for Gro's long-range repression (Chen and Courey, 2000).

MAPK phosphorylation of Yan and Capicua (Cic), two *Drosophila* transcriptional repressors, leads to their nuclear export and degradation (Jimenez *et al*, 2000; Tootle *et al*, 2003); whether Gro is similarly affected is currently unknown. A future challenge would be to elucidate the precise molecular mechanism by which phosphorylation of Gro by MAPK downregulates its repressor potential, that is, does this modulation have an impact on Gro/TLE's homo-oligomerisation, subnuclear localisation, stability, or its interactions with other proteins such as DNA-bound repressors, histones or histone deacetylases?

## ANTAGONISM BETWEEN THE EGFR AND NOTCH PATHWAYS: THE GROUCHO CONNECTION

The long-standing antagonistic relationship between the EGFR and Notch signalling pathways, and the opposing effects exerted by these signal transduction cascades, have been well documented in various developmental settings and organisms (Sundaram, 2005), yet the underlying molecular mechanism linking these pathways has remained poorly understood. As Gro/TLE is a well-established constituent of the N pathway, and given that in response to EGFR activation its repressor capacity is attenuated, it is feasible that this corepressor is at the heart of the crosstalk between these two pathways.

Two *Drosophila* developmental processes, in which the antagonism between the two cascades has previously been established and studied, are the prefiguring of the veins in the wing imaginal disc and of the mesothorax bristles in the adult fly. In these settings, the N pathway acts as an anti-vein and anti-bristle determinant and, in both cases, EGFR signalling functions to overcome the influence of the N pathway in a temporally and spatially restricted fashion, allowing veins and bristles to form (de Celis *et al*, 1997; Culi *et al*, 2001). Several lines of evidence now suggest that EGFR activity promotes vein and bristle formation at least in part by phosphorylating Gro and relieving E(spl)-mediated, Gro-dependent repression, and hence interfering with N signalling (Figure 1B). First, the misexpression of the pseudo-phosphorylated Gro-derivative (Gro^DD^), the presumed end product of MAPK signalling *vis-à-vis* Gro, generates similar effects to those seen when the EGFR pathway is overactivated, that is, extra vein material and supernumerary bristles are formed (Hasson *et al*, 2005). Second, the overexpression of Gro^AA^, the nonphosphorylatable derivative of Gro, renders the N pathway refractory to antagonistic EGFR activity, and the phenotypes caused by the misexpression of Gro^AA^ are comparable to those observed when the Notch pathway is ectopically activated, that is, loss of vein material and of bristles (de Celis *et al*, 1997; Culi *et al*, 2001; Hasson *et al*, 2005).

These results strongly suggest that some of the crosstalk between the N and EGFR pathways takes place at the level of the Gro/TLE corepressor. Crosstalk between these pathways could, of course, also occur at other levels. Significantly, MAPK regulation of Gro, which is at the junction between the two signalling cascades, provides an effective molecular mechanism that enables EGFR signalling to feed into the N cascade and to impinge on N transcriptional output.

## ATTENUATION OF GROUCHO-MEDIATED REPRESSION BY EGFR SIGNALLING: POSSIBLE BIOLOGICAL IMPLICATIONS

The downregulation of Gro's repressor activity by EGFR signalling (and, likely, by other RTK pathways) has several possible biological implications. Below, we elaborate on three of these:

### Coordinated derepression of a large array of genes

**Figure 2:**
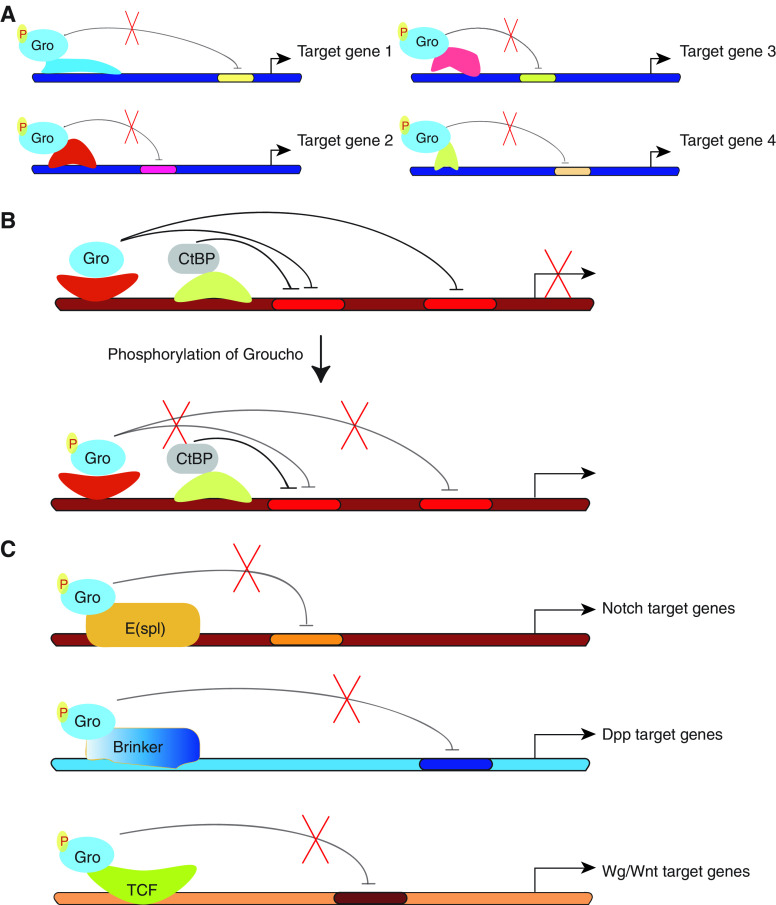
Attenuation of Groucho-mediated repression by RTK signalling: possible implications. (**A**) Coordinated derepression of a large array of genes. (**B**) Switching between modes of transcriptional regulation. (**C**) Groucho integrates RTK signaling with multiple pathways.

RTK signalling effectively alters cell fates, a phenomenon that entails eliciting extensive changes to gene expression programmes involving the transcription of numerous genes (e.g. Roberts *et al*, 2000), yet to date only a few transcription factors that are regulated by MAPK phosphorylation are known. How can such a dramatic change in gene expression profiles be achieved? Gro/TLE is a global corepressor, which acts together with manifold DNA-binding repressors, so it makes an ideal target for EGFR regulation. We propose that relief of Gro-dependent gene silencing, in response to RTK signals in distinct developmental settings, permits the coordinated derepression of a considerable number of genes giving rise to broad changes in gene expression profiles (Figure 2A).

Wing vein patterning illustrates the above point. It turns out that besides the E(spl) proteins, there are at least two other Gro-dependent repressors that obstruct wing vein formation, namely Brk and Cic (Figure 1B; Roch *et al*, 2002; Cook *et al*, 2004). By targeting Gro, the coregulator that is shared between them, EGFR signalling can antagonise the entire group of repressors simultaneously, precluding them in a spatially and temporally regulated manner from exerting their repressor function over vein-making realisator genes. In this way, there is no need to independently regulate each and every anti-vein repressor individually.

### Switching between modes of transcriptional regulation

The downregulation of Gro activity by RTK signalling may be a prerequisite for the transition from gene repression to activation. An increasing number of transcription factors appear to be bimodal in nature, in that on the one hand they act as Gro-dependent repressors, yet on the other hand they also activate gene expression in a context-dependent manner. Strikingly, the Gro-recruitment motifs in some of these transcription factors do not conform to any of the previously defined Gro-binding motifs or, in others, they bind Gro with a lower affinity. An elegant study of Lozenge, a factor belonging to this latter group, showed that when its weak Gro-recruitment motif (the tetrapeptide WRPY) is changed to a stronger Gro-binding motif (WRPW), this protein no longer alternates between its two modes of gene regulation; rather, it now behaves as a constitutive repressor (Canon and Banerjee, 2003). Conversely, and along this same line, we propose that in cells in which the available pool of ‘active’ Gro is lowered in response to RTK signalling, Lozenge and other bi-functional factors containing relatively weak Gro-binding domains will no longer repress, tilting the balance towards transcriptional activation.

RTK signalling could also be at the basis of the switch between long- and short-range repression. It appears that many repressors possess more than one repression domain. Brk and Su(H), for example, rely both on Gro as well as on a second corepressor called CtBP for turning off gene expression (Hasson *et al*, 2001). Gro and CtBP act qualitatively differently, in that Gro mediates repression over long distances (>1 Kb), whereas CtBP functions at short range (100–150 bp) (Figure 2B; Zhang and Levine, 1999). We propose that phosphorylation is a means of abating Gro-mediated repression, allowing repression that is CtBP-dependent (by Brk, Su(H) and/or other repressors) to dominate (Figure 2B). The necessity for such a mechanism is stressed by the fact that most promoters in multicellular organisms are compound, comprised of several stage- and tissue-specific enhancers that contain multiple binding sites for an assortment of transcriptional regulators. Gro-dependent repression should have a regional, dominant effect on adjacent enhancer elements, whereas CtBP acts locally. Thus, RTK signalling could be at the basis of the transition between these two modes of repression. In this scenario, MAPK phosphorylation will override Gro's long-range effect and, in this way, unmask CtBP's proximal repressor capability, allowing enhancer autonomy.

In summary, phosphorylation of Gro can provide a dynamic molecular switch that alternates between transcriptional repression and activation, or between different modes of repression.

### Groucho integrates RTK signalling with multiple pathways

RTK pathways have frequently been shown to interact with other signal transduction pathways, consistent with extensive dialogues taking place between signalling cascades, yet the precise level(s) at which these pathways interconnect have remained largely elusive (Voas and Rebay, 2004). In a like manner to the case of the N and EGFR pathways, Gro/TLE could be a focal point between RTKs and other pathways. As mentioned above, Gro/TLE-dependent repressors function not only downstream of N, but also as effectors of the Dpp/TGF-*β* and Wg/Wnt pathways. The regulation of Gro by RTK signalling is expected to similarly affect the transcriptional output of these cascades (Figure 2C) (Hasson *et al*, 2005; our unpublished data).

Needless to say, crosstalk between pathways also takes place at other focal points. As mentioned above, target promoters frequently integrate distinct signals in the nucleus. A nice example for this is the expression of *D-Pax2* in the *Drosophila* eye, that depends on the combined binding of effectors of both the N and EGFR pathways, together with tissue-specific transcription factors (Flores *et al*, 2000). Some of the intersections between signalling cascades might also occur via mutual cytoplasmic components. For instance, the Shaggy/GSK3 kinase is shared by both the Wg and the Hedgehog pathways (Jia *et al*, 2002).

## CONCLUDING REMARKS

A general coregulator such as Gro/TLE makes a perfect integrator of inputs from RTK signal transduction cascades with multiple signalling networks. Remarkably, post-transcriptional phosphorylation also modulates the activity of several other global cofactors. For example, CtBP activity is modified by HIPK2 phosphorylation (Zhang *et al*, 2003), and HDACs such as Rpd3 have also been shown to be phosphorylated by MAPK in response to cellular stress (De Nadal *et al*, 2004). Thus, it is conceivable that, in addition to Gro, other widely used cofactors play a similar integrative role in cell signalling. It is also likely that similar interactions between RTKs and other pathways exist in vertebrates in general, and in neoplastic cells in particular, at the level of Gro/TLE and other general coregulators.
